# pLAST—a tool for rapid comparison and classification of bacterial plasmid sequences

**DOI:** 10.1093/bioinformatics/btag574

**Published:** 2026-07-29

**Authors:** Kamil Krakowski, Malgorzata Orlowska, Kamil Kaminski, Dariusz Bartosik, Stanislaw Dunin-Horkawicz

**Affiliations:** Institute of Evolutionary Biology, Faculty of Biology, Biological and Chemical Research Centre, University of Warsaw, Zwirki i Wigury 101, Warsaw 02-089, Poland; Institute of Evolutionary Biology, Faculty of Biology, Biological and Chemical Research Centre, University of Warsaw, Zwirki i Wigury 101, Warsaw 02-089, Poland; Institute of Evolutionary Biology, Faculty of Biology, Biological and Chemical Research Centre, University of Warsaw, Zwirki i Wigury 101, Warsaw 02-089, Poland; Department of Bacterial Genetics, Institute of Microbiology, Faculty of Biology, University of Warsaw, Miecznikowa 1, Warsaw 02-096, Poland; Institute of Evolutionary Biology, Faculty of Biology, Biological and Chemical Research Centre, University of Warsaw, Zwirki i Wigury 101, Warsaw 02-089, Poland; Department of Protein Evolution, Max Planck Institute for Biology Tübingen, Max-Planck-Ring 5, Tübingen 72076, Germany

## Abstract

**Motivation:**

The increasing number of fully sequenced bacterial plasmids being annotated and catalogued has prompted the development of computational tools for comparing and classifying them. Existing approaches typically compare full-length DNA sequences (e.g. Mash, BLASTn, and ANI-based methods) or translated open reading frames (ORFs) (e.g. DIAMOND), with plasmid-level scores obtained by aggregating ORF-to-ORF similarities; however, they are either restricted to closely related plasmids or become computationally demanding in large-scale analyses.

**Results:**

We describe pLAST (plasmid Language Analysis and Search Tool), a plasmid-search tool built using word2vec representations of protein-family content informed by local genomic context. Benchmarks indicate that pLAST outperforms nucleotide-based methods and performs comparably to DIAMOND in identifying functionally similar plasmids and compared with the widely used Mash, it achieves 26% and 24% improvements in detecting shared mating-pair formation system type and relaxase type, respectively. This performance scales to database searches across hundreds of thousands of sequences, as demonstrated using the precomputed PlasmidScope collection of ∼750 000 plasmids. Beyond global similarity, pLAST also returns per-ORF plasmid-plasmid alignments, enabling detection of shared functional modules.

**Availability and implementation:**

pLAST is freely accessible as a web server at https://plast.lbs.cent.uw.edu.pl/ or https://plast.lbs.biol.uw.edu.pl/ and available as a Python module along with a precomputed database at https://github.com/labstructbioinf/pLAST for customized analysis.

## 1 Introduction

Plasmids are extrachromosomal replicons that drive horizontal gene transfer, spreading antibiotic- and metal-resistance genes as well as virulence factors (Dewan and Uecker 2023, Coluzzi and Rocha 2025). Given their biological and evolutionary significance, dedicated plasmid databases are expanding rapidly. The PLSDB database has increased from ∼34k non-redundant entries in 2021 to ∼72k in the 2025 update (Molano *et al.* 2025). Non-dereplicated resources contain even more sequences, with IMG/PR and PlasmidScope databases hosting ∼700k and ∼852k plasmid sequences, respectively, including plasmids reconstructed from metagenomes (Camargo *et al.* 2024, Li *et al.* 2025).

Searching these resources relies on tools that compare a query plasmid against database entries. Common options include DNA-based methods such as Mash (Ondov *et al.* 2016), BLASTn (Altschul *et al.* 1990) or Average Nucleotide Identity (ANI) methods like skani (Shaw and Yu 2023) or fastANI (Jain *et al.* 2018), as well as protein-based approaches leveraging ORF-to-ORF similarities returned by tools like DIAMOND (Buchfink *et al.* 2015). DNA methods typically compare whole plasmid nucleotide sequences to assess global similarity between plasmids (Schmartz *et al.* 2022, Camargo *et al.* 2024), whereas ORF-based frameworks aggregate ORF-to-ORF hits for gene-content comparison and module detection (Suzuki *et al.* 2020, Teixeira *et al.* 2023). Other approaches focus primarily on plasmid typing, clustering, or rearrangement-aware relationships, including COPLA (Redondo-Salvo *et al.* 2021), mge-cluster (Arredondo-Alonso *et al.* 2023), and pling (Frolova *et al.* 2024), rather than on ranking database entries by similarity to a query plasmid.

The application of skani, fastANI, Mash, or BLASTn to plasmid comparison has key limitations: first, they operate on DNA sequences, which diverge faster than protein sequences, so for distant relationships, the homology signal often falls below detection; second, plasmids are modular and often mosaic, so shared sequence is frequently discontinuous, and accessory regions and mobile elements can distort global similarity measures (Redondo-Salvo *et al.* 2020); third, methods relying on sequence alignment (e.g. BLASTn) may not explicitly account for sequence circularity, which may in some cases reduce similarity estimates between otherwise highly similar plasmids, a problem explicitly targeted by some methods (Fernandes *et al.* 2009, Grossi *et al.* 2016, Ayad and Pissis 2017).

These problems, highlighted in discussions on plasmid similarity metrics (Matlock *et al.* 2024), are partly mitigated by ORF-based methods, which are generally more robust for detecting distant homology. Among such approaches, profile HMMs provide high sensitivity for targeted annotation of specific protein families, but are computationally demanding. Faster yet less sensitive methods, such as DIAMOND, enable broader searches, but still incur substantial computational costs in all-against-all ORF comparisons at the scale of databases like PlasmidScope. Moreover, conventional methods that aggregate independent ORF-to-ORF comparisons do not explicitly model circularity or modular architectures, although comparing ORFs independently makes results largely insensitive to sequence rotation and gene order. However, the downside is that ignoring gene order discards synteny, an evolutionarily informative signal; in addition, protein-level matches that do not consider genomic context can be confounded by paralogs, especially those originating from recent gene duplications (Maida *et al.* 2014).

Here, we describe pLAST, which applies a word2vec model, originally developed for natural language processing, to plasmids treated as ‘sentences’ of ‘words’ (protein families), thereby learning local gene-neighbourhood relationships and enabling functionally meaningful database searches and scalable clustering.

## 2 Materials and methods

### 2.1 Dataset preparation

More than 750 000 plasmids were retrieved from the PlasmidScope database (accessed May 2026) and annotated with MOB-typer (v3.1.9), a component of MOB-suite (Robertson and Nash 2018). The dataset contained ∼25 million proteins, of which ∼10.6 million had unique sequences. These were clustered with MMseqs2 (18.8cc5c) (Steinegger and Söding 2017) using the parameters (-s 7.5 --cluster-mode 1 --cov-mode 0 --min-seq-id 0.3 -c 0.8), yielding nearly 2 million unique clusters.

### 2.2 Model training

PlasmidScope plasmids were encoded as sequences of ORFs, represented by MMseqs2 cluster identifiers. To avoid dependence on the arbitrary start coordinate, during dataset creation each ORF chain was randomly circularly permuted (only for the circular records) and, with 50% probability, reversed in order. Next, a word2vec model was trained for the full PlasmidScope dataset with ORFs encoded as MMseqs2 clusters. To this end, Gensim (4.3.3) (Řehůřek and Sojka 2010) was used with the following parameters: CBOW architecture (sg = 0), vector dimension = 64, context window size = 4, min_count = 2, and negative sampling with negative = 15. The model was trained for 30 epochs, producing embeddings for every unique MMseqs2 cluster ID present after min-count filtering; the effective vocabulary size was 778 475.

Moreover, a placeholder ‘blank-ORF’ vector was computed by averaging all ORF embeddings. Subsequently, plasmid embeddings were computed as the mean of their ORF embeddings; ORFs missing a cluster assignment or whose assigned cluster identifier was missing in the given model received the placeholder embedding.

### 2.3 Functional benchmark

In the benchmark, we focused on potentially conjugative or mobilizable plasmids by selecting PlasmidScope records originating from RefSeq or GenBank and annotated by MOB-suite with either a relaxase type or a mating-pair formation (MPF) system type. MOB-suite primary and secondary clusters (Robertson *et al.* 2020) were used during benchmark set construction to reduce redundancy, increase diversity within the selected plasmid pool, and thus make the benchmark more challenging. Within each MOB-suite primary cluster of the resulting 5360 plasmids, ∼10% were randomly sampled, with at least one representative retained per primary cluster where possible and representatives from distinct MOB-suite secondary clusters prioritized, yielding 1288 plasmids from 1075 primary clusters. Two hundred plasmids were then randomly selected as queries, with at most one query per MOB-suite primary cluster; the full sampled set of 1288 plasmids served as the database, with self-hits excluded during subsequent ranking.

The 200 queries were used to search the benchmark database with pLAST, where similarity was computed as the cosine between unit-normalized plasmid embeddings, and with five baselines: (i) DIAMOND 2.1.12 (E-value cut-off = 1e-3 and ‘--more-sensitive’ option) scored as the average of per-ORF best-hit bit scores over the query ORFs (before aggregation, greedy one-to-one matching of query and target ORFs within each plasmid pair and ordering hits by higher bit score and lower E-value was applied), (ii) Mash 2.3 (*k* = 19, sketch size = 10^5), with similarity defined as 1 − distance after filtering hits with *P*-value ≤ 1e-3 and at least 100 shared hashes, (iii) skani 0.3.2, (iv) fastANI 1.34, both scored using ANI/100, and (v) BLASTn 2.16.0+ (E-value ≤ 1e-3), with similarity defined as ANI × AF, where ANI is the average nucleotide identity over uniquely covered positions in the query, and AF (alignment fraction) is defined as the smaller of the uniquely covered query and subject lengths divided by the length of the shorter sequence, i.e. the fraction of the shorter sequence that is aligned in both sequences. For methods that did not report a hit for a given query-database pair, the pair was retained in the ranking and assigned a similarity score of 0, effectively placing it after all reported hits for that query.

**Figure 1:**
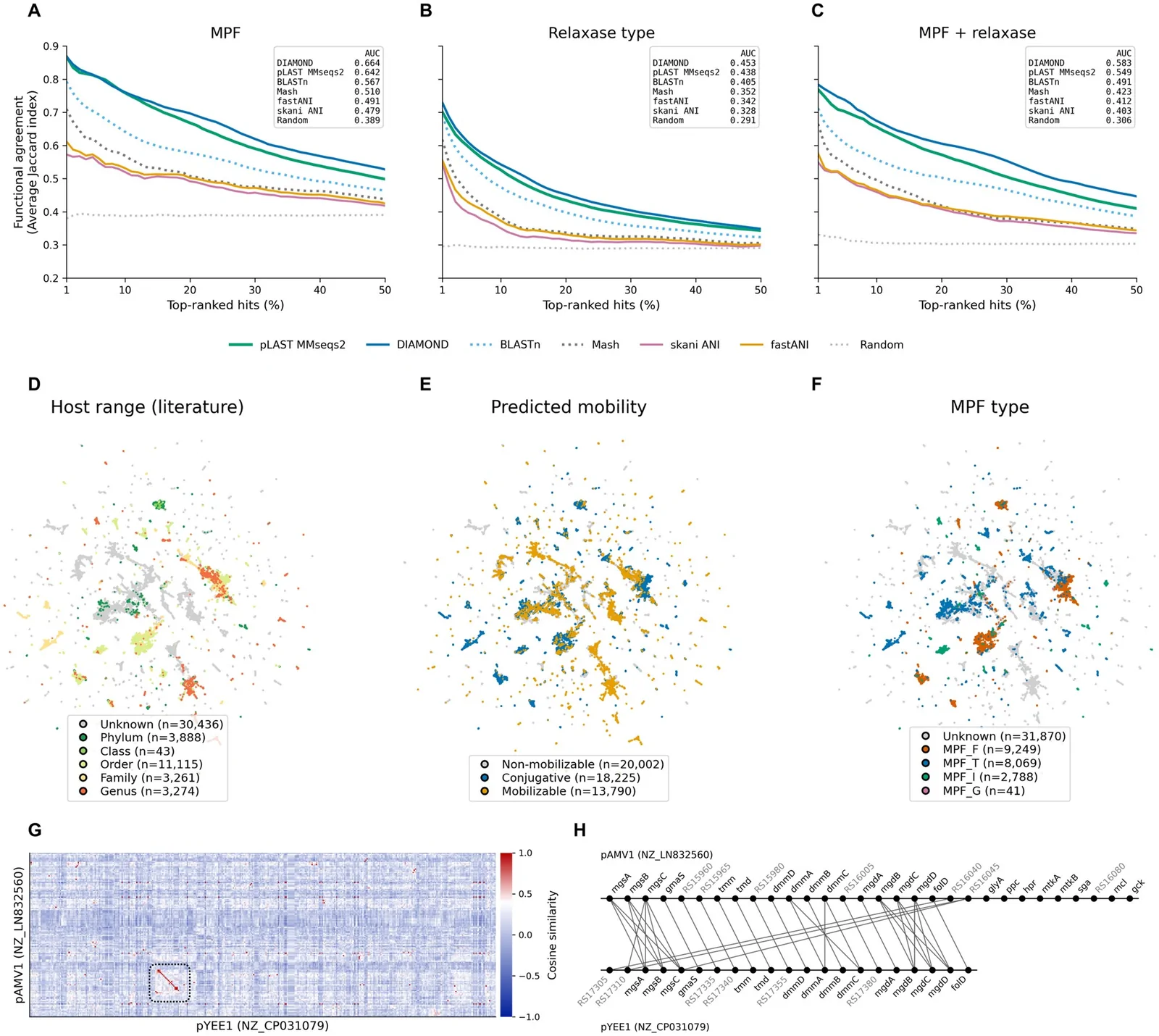
Performance of pLAST and other methods in detecting functionally similar plasmids. Three benchmark categories are evaluated: (A) MPF (mating-pair formation) type, (B) relaxase type, and (C) combined MPF and relaxase annotations. The *x*-axis shows the percentage P of top-ranked database hits retained and the *y*-axis shows the mean Jaccard index between each query’s annotations and those of its top P% hits, averaged across the eligible queries. AUC values are shown in each inset. Panels D–F display UMAP projections of pLAST embeddings for ∼52 000 plasmids, coloured by host range (D), predicted mobility (E), and MPF type (F) from MOB-suite. (G) Per-ORF cosine similarity matrix between the plasmids pAMV1 (NZ_LN832560) and pYEE1 (NZ_CP031079). (H) Zoomed-in view of the dotted box in (G), highlighting a multi-gene syntenic region; dots are ORFs and connecting lines indicate ORF pairs with embedding cosine similarity ≥0.6.

For each query, we computed the mean Jaccard index between its MOB-suite annotations and those of the top P% most similar database hits, where P ranged from 1% to 50%, and then averaged these scores across queries. We performed this analysis separately for two annotation categories, i.e. the relaxase type and MPF system type, and for the combined MPF + relaxase annotation. Queries and database hits lacking an annotation in the evaluated category were excluded for that category. This yielded performance curves and area-under-the-curve (AUC) scores (Fig. 1), which we used to compare the pLAST model to DIAMOND, Mash, skani, fastANI, and BLASTn.

## 3 Results

pLAST was built by training a word2vec model on plasmids encoded as sequences of ORFs, each represented by the identifier of the protein family it belongs to. These protein families were derived by clustering ∼10.6 million unique protein sequences translated from ORFs of ∼750 000 plasmids with MMseqs2. For a given plasmid, the trained model computes its numerical representation (embedding), which captures ORF content using context-informed protein-family vectors along the plasmid. These embeddings can then be quickly compared across plasmids to estimate their similarity. Because pLAST operates on protein-family identifiers rather than directly on amino acid sequences, its score reflects similarities in protein-family content and local genomic context, not protein sequence similarity across the plasmid. Thus, a high pLAST score indicates that two plasmids have similar context-informed protein-family compositions, but does not necessarily identify the plasmid whose corresponding ORFs have the highest amino acid sequence similarity to those of the query.

To evaluate pLAST performance, we benchmarked it against DNA sequence-based methods (BLASTn, Mash, fastANI, and skani) and a protein sequence-based method (DIAMOND) for their ability to retrieve functionally similar plasmids. We constructed a diverse, nonredundant benchmark database of 1288 conjugative or mobilizable plasmids and used 200 plasmids from this set as queries to assess how well each method retrieved plasmids sharing relaxase and MPF system types as defined by MOB-suite (Fig. 1A and B). Each of these two functional categories poses a distinct challenge: relaxase type is determined by a single locus, whereas MPF is a large, multi-gene module spanning many ORFs and often arranged in operons with mosaic variation. We also considered the combination of the two, evaluating agreement across the combined relaxase and MPF type annotations (Fig. 1C).

Across functional categories, pLAST using MMseqs2-defined protein families achieved performance comparable to DIAMOND, followed by BLASTn, Mash, and ANI-based methods (skani and fastANI; Fig. 1). In this benchmark, DNA-based methods underperformed because conservation of protein-level functional modules is not well captured at the nucleotide level, whether assessed using global k-mer sketch similarity (Mash), local alignments (BLASTn), or ANI-based comparisons (skani and fastANI). By contrast, the protein-based method DIAMOND performed substantially better, as it is more robust to sequence divergence and genomic rearrangements. The comparable performance of pLAST is based on employing protein-level similarity (MMseqs2-defined protein families) with local co-occurrence context captured by the word2vec model.

In terms of retrieval time using precomputed method-specific representations or indices, searching the benchmark database with 200 queries took 2.7 minutes for BLASTn, 47 s for DIAMOND, 28 s for Mash, 13 s for fastANI, and 1.8 s for skani. By comparison, pLAST returned the ranked results from precomputed plasmid embeddings in 0.72 s, offering a 40-fold speed-up over Mash for the retrieval step, while providing performance comparable to the ORF-based method DIAMOND. Comparisons of pLAST representations scale easily to much larger datasets, as demonstrated by effective searches against databases as large as PlasmidScope and by a UMAP projection of ∼52 000 RefSeq plasmids, revealing functionally coherent groups whose mobility gene repertoires are consistent with the reported host range (Fig. 1D–F).

The benchmark (Fig. 1A–C) and the UMAP projection (Fig. 1D–F) described above rely on global plasmid-plasmid similarity scores; however, pLAST also returns ORF-level alignments, enabling the characterization of multi-gene regions shared between plasmids. For example, the methylotrophy islands (MEIs) are modular cassettes enabling the use of single-carbon (C1) compounds as a carbon and energy source. In the pAMV1 plasmid of *Paracoccus aminovorans* JCM 7685 (NZ_LN832560), the MEI comprises modules for substrate uptake and oxidation, THF-linked C1 processing, and assimilation via the serine cycle (Czarnecki *et al.* 2017). Its truncated variant, lacking the serine-cycle module (and thus C1 assimilation), has been reported in the *Paracoccus yeei* CCUG 32053 pYEE1 plasmid (NZ_CP031079; Czarnecki and Bartosik 2019). The pLAST alignment of pAMV1 and pYEE1 (Fig. 1G and H) shows that, despite their low overall similarity, the plasmids share the MEI region; it also reveals differences, including the loss of the serine-cycle module and a circular rearrangement that relocates two ORFs to the beginning of the cassette. The crossed lines observed for some genes in the *mgsABC*, *dmmABC*, and *mgdABCD* modules reflect similarity in genomic context rather than direct amino acid sequence similarity indicative of homology between the corresponding ORFs.

pLAST is available as a web server at https://plast.lbs.cent.uw.edu.pl/ or https://plast.lbs.biol.uw.edu.pl/. The server enables searches against a precomputed PlasmidScope database using entire plasmids or functional modules of interest. The resulting matches can be analysed globally via UMAP projections, such as those shown in Fig. 1D–F, or individually by inspecting pairwise alignments. Together, these features enable rapid and comprehensive characterization of plasmids in the context of the bacterial plasmidome, as well as assessment of the genetic diversity of these replicons and the rearrangements that give rise to their variants.
